# Advances in the application of different imaging techniques in percutaneous lung biopsy

**DOI:** 10.3389/fmed.2026.1732266

**Published:** 2026-05-07

**Authors:** Ziou Zhao, Jie Hou, Muhammad Asad Iqbal, Xin Wang, Hui Zhou, Hui Sun, Xian Wang

**Affiliations:** 1 Department of Ultrasound, Affiliated People’s Hospital of Jiangsu University, Zhenjiang, China; 2 Department of Ultrasound, Women's Hospital, Zhejiang University School of Medicine, Hangzhou, China; 3 Department of Ultrasound, Wuxi Second Hospital of Traditional Chinese Medicine, Wuxi, Jiangsu, China; 4 Department of Pathology, Affiliated People’s Hospital of Jiangsu University, Zhenjiang, China

**Keywords:** angiography, CT, magnetic resonance, percutaneous lung biopsy, ultrasonic

## Abstract

The qualitative diagnosis of lung diseases has always been a difficult problem in clinical treatment, especially in the differentiation of benign and malignant nodules. With the rapid development of imaging technology, percutaneous lung biopsy has become the main means of diagnosing and differentiating lung lesions. For a long time, CT guidance has been the main method of percutaneous lung biopsy, but with the continuous improvement of imaging technology, especially the vigorous development of ultrasound technology, its application in lung diseases is increasing, and the application of contrast-enhanced ultrasound has further improved the diagnostic value of lung diseases. This article reviews the application and progress of various imaging techniques in percutaneous lung biopsy.

## 1 Introduction

At present, lung cancer ranks first among the leading causes of death among cancer patients in China, and its incidence and mortality rates are still increasing year by year. The early onset of peripheral lung cancer is relatively insidious, and the clinical symptoms are mostly not obvious, and most patients are already in the middle and advanced stages when they visit the hospital, and the prognosis is poor. Therefore, early and accurate diagnosis of peripheral lung cancer is of great significance to improve the clinical treatment effect and improve the prognosis of patients (1).

Conventional bronchoscopy has a high detection rate for central lung cancer, but relatively low diagnostic accuracy for peripheral lung cancer. Sputum exfoliation cytology has low specificity and poor sensitivity in diagnosing peripheral lung cancer (2), and its diagnostic accuracy is much lower than that of imaging-guided percutaneous lung biopsy. However, thoracotomy and thoracoscopic lung biopsy are expensive and risky, and are difficult to tolerate in patients with advanced age and poor cardiopulmonary function, and their clinical application is uncommon (3). At present, the diagnosis of peripheral lung lesions is mainly done through percutaneous lung puncture biopsy, which can determine the nature of the mass and greatly improve the diagnosis rate of peripheral lung cancer using Computed Tomography (CT), Positron Emission Tomography/Computed Tomography (PET/CT), and Magnetic Resonance Imaging (MRI). This article reviews the application of ultrasound and ultrasound contrast-enhanced percutaneous lung puncture biopsy in lung diseases.

## 2 CT-guided percutaneous lung biopsy

CT-guided percutaneous lung puncture biopsy is very common in clinical practice and is an important imaging method for diagnosing lung diseases. It has the characteristics of accurate positioning, high spatial resolution and density resolution, and can clearly show the anatomical structure of the lungs, the size, location, morphology of the lesions, and their relationship with surrounding structures. It also has the ability of rapid scanning and rapid image post-processing without overlapping effects, especially for small lesions behind the cardiac shadow and beside the mediastinum (4). Compared with other guidance methods, CT-guided percutaneous lung puncture biopsy is not affected by factors such as lung gas and bones, and can effectively puncture truly valuable diseased tissues, greatly improving diagnostic accuracy.

**Figure 1:**
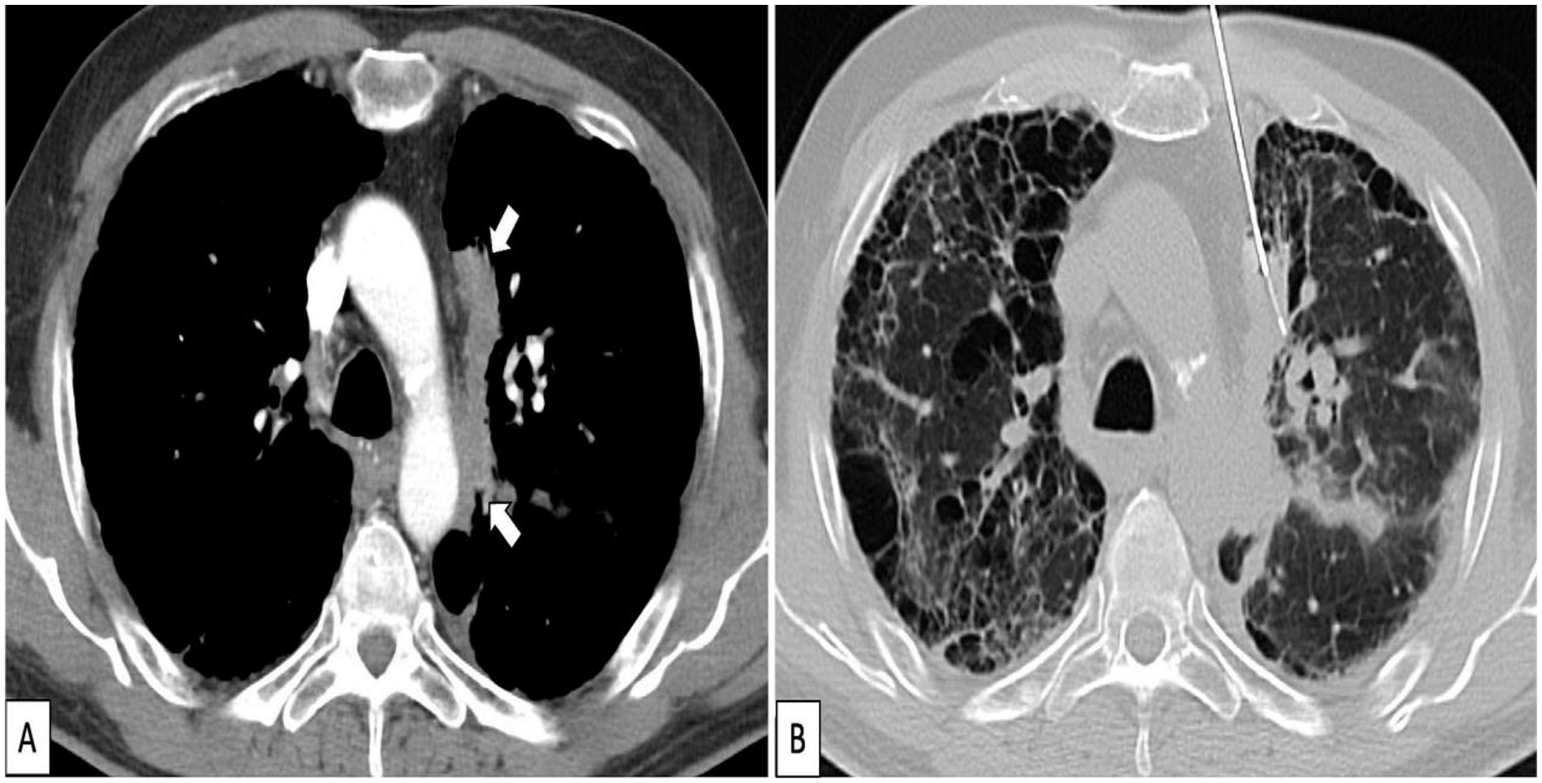
CT-guided percutaneous lung puncture. **(A)** Enhanced CT of the solid tissue adjacent to the left mediastinum (indicated by the arrow). **(B)** The CT-guided biopsy procedure was planned to minimize needle traversal of confluent emphysema by crossing the anterior mediastinum.

For some lesions that are smaller and located in the lower lobe, mediastinum or hilar area, it is difficult to perform a puncture biopsy, so enhanced CT is often needed to understand the blood supply of the lesion, thereby improving the accuracy of diagnosis. Enhanced CT scanning can highlight substantial lesions and clearly show the blood supply inside the lesion to exclude vascular lesions. On the other hand, enhanced CT can distinguish between necrotic and non-necrotic areas of the lesion, and better observe the surrounding structures of the lesion, such as interlobar fissures, bullae etc. (5). Avoiding these areas during puncture and selecting the optimal puncture path can reduce the number of punctures, shorten the procedure time, and decrease the risk of complications, as shown in Figure 1 (6). This can reduce the number of punctures and shorten the puncture time, which can reduce the occurrence of complications to a certain extent. However, for patients with rich blood supply in the lesion and atelectasis, the number of punctures may be increased due to unsatisfactory sampling, which may increase the risk of complications such as bleeding and pneumothorax (7). In major domestic and international guidelines, CT guidance has been established as the first-line recommendation and preferred method for percutaneous lung biopsy due to its high resolution, universal applicability, and standardized procedure, particularly suitable for deep pulmonary parenchymal lesions (8–10).

## 3 Application of PET/CT in percutaneous lung biopsy

The PET/CT is an imaging examination technology that organically combines positron emission tomography (PET) and computed tomography (CT) (11).

**Figure 2:**
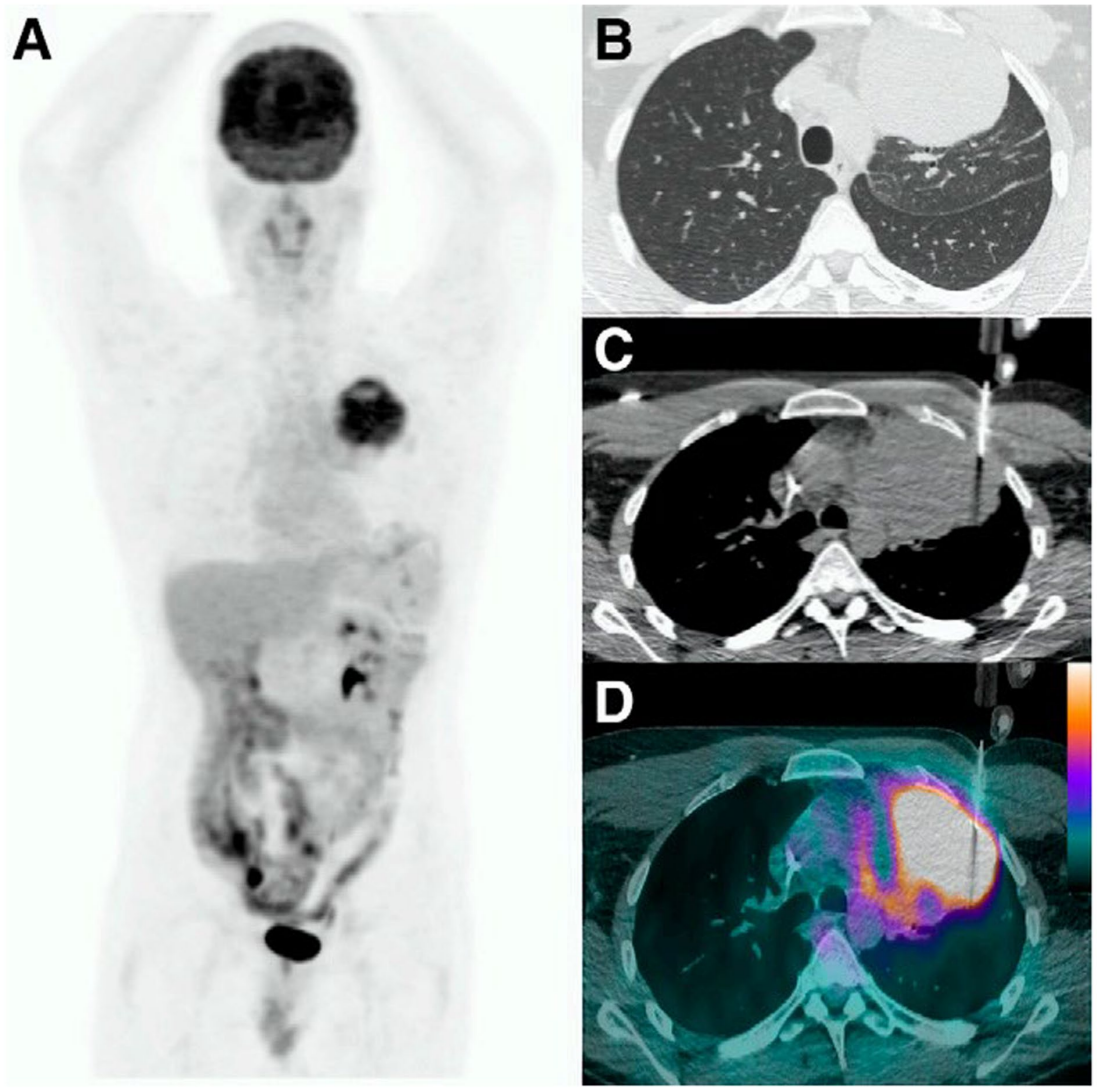
PET-CT fusion image confirms that the puncture reaches the high metabolic. **(A)** Maximum-intensity-projection PET image. **(B)** Transaxial CT lung window. **(C)** Transaxial CT mediastinal window (intraprocedure). **(D)** Transaxial ^18^F-FDG PET/CT image (intraprocedure CT image fused with previously acquired PET/CT image).

The PET/CT can provide accurate and intuitive guidance for percutaneous lung puncture, improve the success rate of puncture and reduce damage to surrounding normal tissues. Conventional CT, especially low-dose scanning CT-guided percutaneous lung biopsy, tends to have low image density resolution, and the positive rate of puncture may be low for some lung lesions with atypical morphology or with necrosis, obstructive atelectasis, and inflammation. PET/CT can distinguish between solid and necrotic areas of the lesion, and guide the needle to avoid the necrotic area and directly draw from tumor tissues with high metabolic activity, as shown in Figure 2 (12). As a result, the success rate and positive rate of biopsy are greatly improved. PET/CT fusion images can also reflect the pathophysiological status of the lesion and the morphological and structural characteristics of the lesion at the same time, especially in the diagnosis of lung peripheral lesions, PET/CT-guided needle biopsy has obvious advantages. Furthermore, with the advancement of precision oncology, biopsies are no longer limited to determining benign or malignant nature but also require obtaining sufficient tumor tissue for molecular pathological testing (e.g., gene mutations, PD-L1 expression, etc.). PET/CT, due to its ability to identify metabolically active tumor regions, helps avoid sampling necrotic or low-activity tissues, thereby enhancing the success rate and reliability of molecular testing (13).

However, compared with traditional contrast-enhanced CT, PET/CT has the disadvantage that it cannot show the relationship between the lesion and the adjacent blood vessels, and it is easy to damage the blood vessels and lead to complications when the puncture is located in the hilum or the lesion with more blood vessels. In addition, some inflammatory lesions, such as active tuberculosis, can appear on PET images with high uptake of 18F-FDG similar to malignant tumors, leading to diagnostic errors and may lead patients to undergo unnecessary needle biopsy or overtreatment (14). At the same time, the high cost of PET/CT examination equipment, coupled with the cost of needle biopsy, makes the overall cost of PET/CT-guided percutaneous lung biopsy high, which limits the application of PET/CT to a certain extent. According to the ACR Appropriateness Criteria, PET/CT guidance is not considered a routine first-line method. Instead, it is selectively recommended for complex lesions that are difficult to characterize with conventional CT, such as those with necrosis, high metabolic heterogeneity, or post-treatment residual masses. Its purpose is to localize areas of high metabolic activity, optimize sampling, and provide higher-quality specimens for molecular pathological testing (15).

## 4 Application of MRI in percutaneous lung biopsy

**Figure 3:**
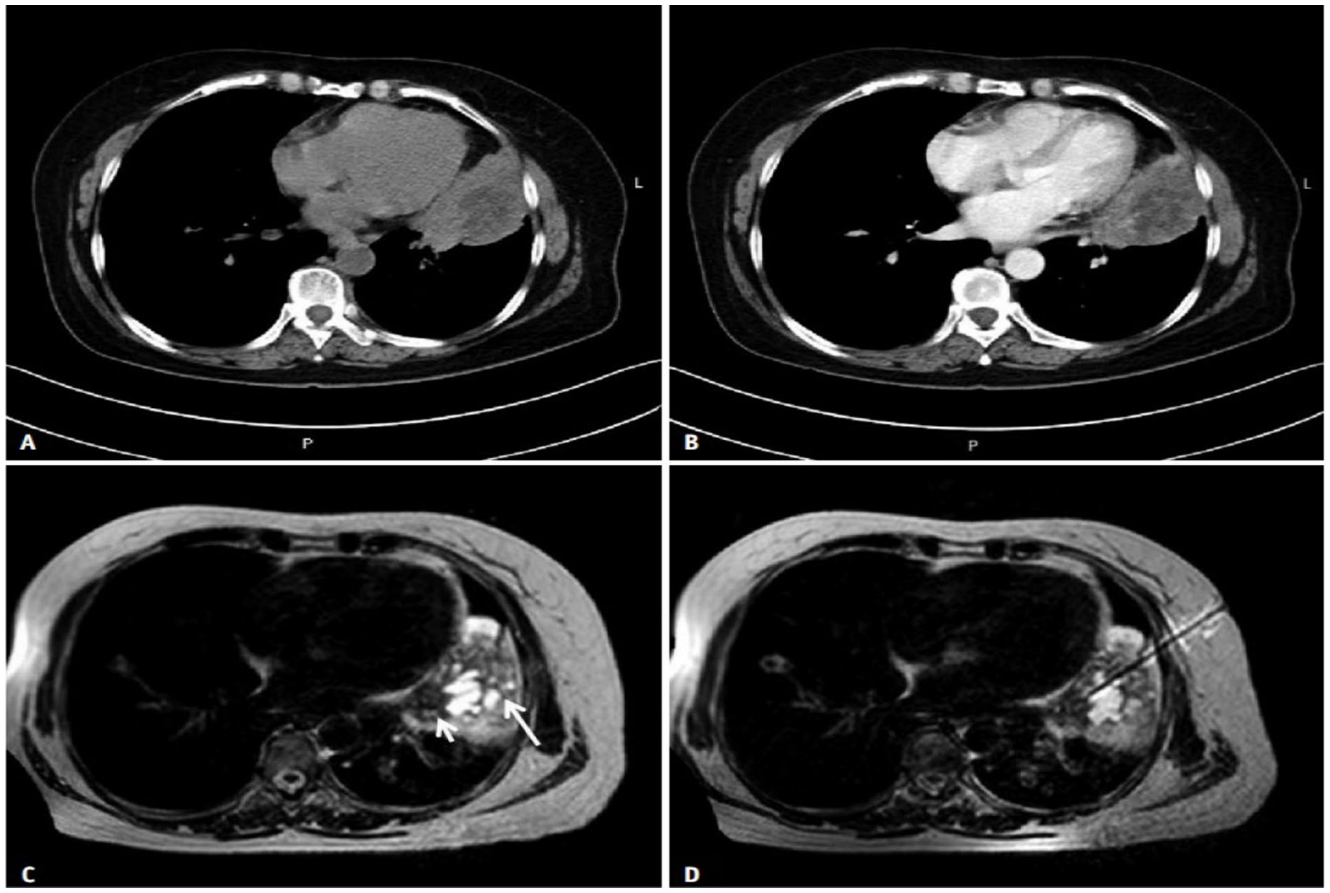
MRI-guided lung puncture. **(A,B)** Non-contrast-enhanced and contrast-enhanced CT did not allow reliable differentiation of tumors from the adjacent atelectasis. **(C)** Fast T2-weighted imaging-turbo spin echo morphological MRI performed at the same level shows post-obstructive atelectasis (long arrow) with a higher signal intensity than the central tumor (short arrow), reflecting a higher water content. **(D)** MRI-guided puncture needle in the lesion.

As a new technology, MRI-guided percutaneous lung biopsy has relatively few clinical research reports. In recent years, with the rapid development of MRI interventional technology, MRI-guided technology without ionizing radiation has been applied to many fields. MRI-guided percutaneous lung biopsy can provide rich tissue contrast imaging information without serious complications, and can be widely used in clinical practice (16). Low-field open interventional MRI guidance is a safe and accurate technology for diagnosing lung lesions: (1) MRI scans images can clearly show lung texture, the size, shape and boundaries of lung lesions; (2) MRI has the advantages of multi-plane imaging capability, excellent soft tissue contrast, good spatial resolution and no ionizing radiation without contrast agents (17); (3) MRI can obtain images in a short time by using breath-holding technology to guide puncture biopsy; (4) MRI open device can get close to the patient well and have enough space for interventional surgery. (5) ipath200 optical tracking system can automatically track the puncture plane. The system dynamically displays the needle path in real time and provides precise positioning for puncture, as shown in Figure 3 (17). The scanned image on the display can be updated so that the operator can adjust the needle insertion direction in time, which not only improves the accuracy of diagnosis but also avoids complications and unnecessary injuries caused by repeated needle punctures (18).

MRI-guided percutaneous lung puncture biopsy also has its shortcomings: (1) MRI scanning time is relatively long, and some patients with poor conditions may find it difficult to hold their breath for a long time, which will affect the imaging effect and thus the accuracy of the puncture; (2) The image update of MRI fluoroscopy technology has a delay time, which will affect the continuity of the puncture; (3) The quality of MRI’s near-real-time sequence images is poor, especially the display of small lung nodules (19). In response to these challenges, significant progress has been made in MRI-guided technology in recent years. The application of rapid imaging sequences and compressed sensing techniques has greatly shortened image acquisition time and improved temporal resolution, contributing to near real-time imaging guidance (20). Meanwhile, advanced respiratory gating and motion compensation technologies can reduce respiratory motion artifacts and enhance the visibility of small lesions. Additionally, multiparametric MRI, such as diffusion-weighted imaging, provides functional information that helps differentiate between benign and malignant lesions and identify active regions, thereby optimizing the selection of biopsy targets (21). Given its absence of ionizing radiation and superior soft tissue contrast, MRI-guided percutaneous lung biopsy is often an important option in specific scenarios. It is particularly suitable for patients who must absolutely avoid radiation (such as pregnant women and adolescents) or for special cases where the lesion is adjacent to major mediastinal blood vessels (15).

## 5 Ultrasound-guided percutaneous lung biopsy

**Figure 4:**
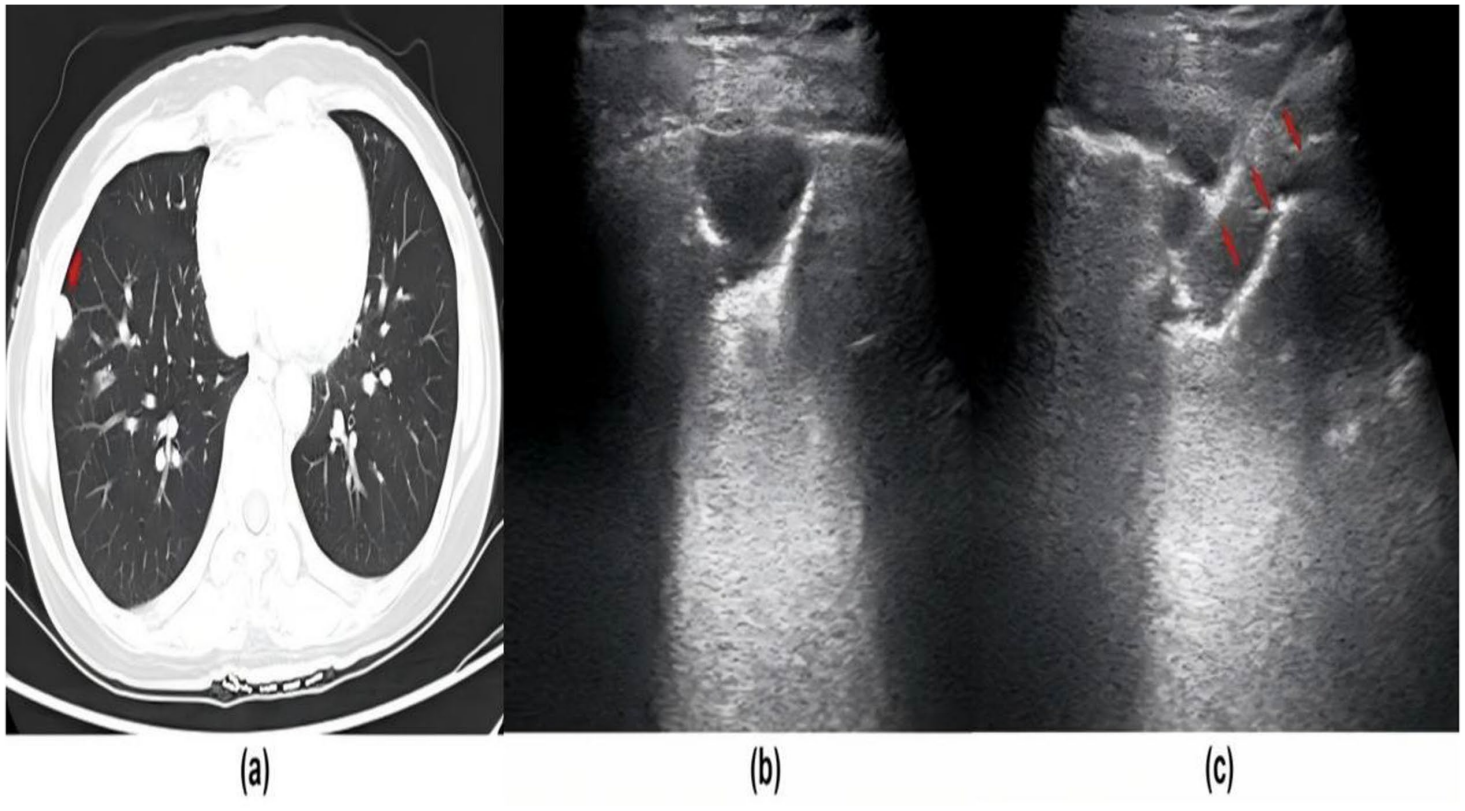
Ultrasound-guided lung puncture. **(a)** CT image of the peripheral pulmonary lesion (red arrow) located at the right upper lobe. **(b)** Baseline ultrasound showed the irregular and hypoechoic lesion (red arrow). **(c)** A 16-G needle was used to perform biopsy for the lesion. The needle tract (red arrow) could be seen on ultrasound.

Although the diagnostic accuracy of peripheral lung masses has been greatly improved with the development of enhanced CT, MRI and other imaging technologies, the identification of the cause and nature of peripheral lung masses is still difficult because peripheral lung masses with different pathologies often have similar imaging manifestations. In recent years, with the development of ultrasound technology, ultrasound-guided percutaneous lung mass puncture has been widely used in clinical practice, as shown in Figure 4 (22). In particular, lung masses near the pleura are close to the chest wall and are not affected by lung gas. The lesion display rate of ultrasound is similar to that of CT (22).

Ultrasound-guided percutaneous lung mass puncture biopsy has the following advantages: (1) Ultrasound-guided puncture can dynamically observe the lesion in real time and adjust the direction and depth of the needle at any time, which makes up for the lack of synchronization between CT guidance and puncture and improves the safety of puncture. (2) Ultrasound can scan the lesion in all directions, which is conducive to selecting the best needle insertion path and improving the accuracy of diagnosis. (3) Color Doppler ultrasound can clearly show the blood flow distribution in the lesion, avoiding damage to blood vessels and surrounding important tissues (23). Previous literature reports show that the complication rate of ultrasound-guided percutaneous lung puncture biopsy is lower than that of CT-guided percutaneous lung puncture biopsy. The reason is that even if the lesion has invaded the lung surface, as long as there is no interference from lung tissue and gas in the outer edge, the normal lung parenchyma will not be damaged during most of the puncture process, so the complication rate is relatively low (24).

Compared with CT, ultrasound-guided percutaneous lung puncture biopsy is less invasive and safer, inexpensive, real-time dynamic, and radiation-free. It has low requirements on body position and can be performed in a sitting position even for patients who cannot lie flat. It has a high diagnostic accuracy rate (25). However, ultrasound is affected by lung gas and can only select lesions at the edge of the lung. It cannot puncture the hilum, mediastinum, or deeper lesions. Secondly, if the lesion contains liquefied necrosis, it is easy to puncture the liquefied necrotic tissue under conventional ultrasound, which reduces the accuracy of diagnosis (26). The BTS guidelines state that ultrasound guidance should be prioritized for peripheral pulmonary lesions abutting the chest wall. The Chinese multidisciplinary expert consensus similarly designates it as the preferred method for such cases. Due to its advantages of real-time imaging, absence of radiation, and low cost, ultrasound has become the first-line recommended method for biopsy of such lesions (8, 27).

## 6 Application of contrast-enhanced ultrasound (CEUS) in percutaneous lung biopsy

Since conventional ultrasound is easily interfered by gas, its application in lung lesions is mainly limited to peripheral lesions and central lesions with obstructive atelectasis. Color Doppler ultrasound often displays incorrect blood flow information inside the lesion due to the lack of blood vessels or interference from heart beats, which affects the accuracy of diagnosis (28). It is generally believed that extremely low-echo or no-echo areas are necrotic tissue of the lesion, and low-echo, isoechoic and high-echo areas are effective tissue. However, sometimes ultrasound images show low to high-echo areas, but the puncture biopsy results are necrotic tissue. It can be seen that conventional ultrasound cannot truly reflect whether the lesion is necrotic tissue or effective tissue, nor can it clearly distinguish the adjacent relationship of surrounding large blood vessels, resulting in certain false negatives and sampling failures (29).

**Figure 5:**
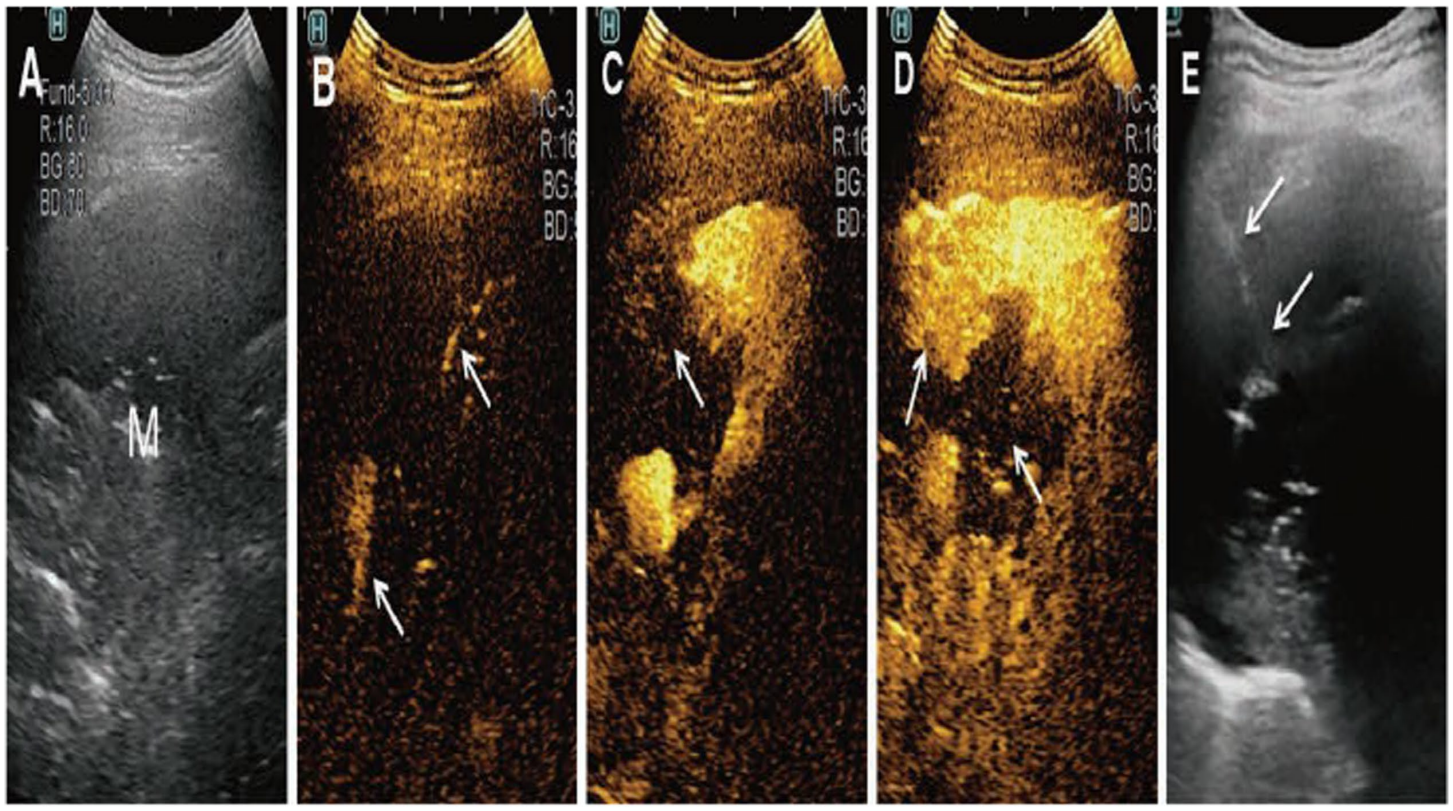
Lung puncture under contrast-enhanced ultrasound. **(A)** Conventional ultrasound showed a mass lesion (M). **(B)** Contrast agent reaching lung tissues. **(C)** Contrast agent reaching lesions tissues. **(D)** The mass was completely enhanced. The enhancement mode is centripetal, non-uniformly. **(E)** Ultrasound-guided puncture.

CEUS can not only show the internal structure of the lesion, reflect the effective tissue of the lesion, and distinguish the adjacent relationship between the lesion and the surrounding large blood vessels, but also help screen biopsy indications and reduce unnecessary damage to patients (29). This further improves the accuracy of ultrasound-guided percutaneous lung puncture biopsy, as shown in Figure 5 (30). Studies have shown that the success rate of ultrasound-guided percutaneous lung puncture after CEUS can be increased to 97.62% (30).

The CEUS can also be used to preliminarily differentiate between benign and malignant lung lesions. Studies have shown that benign lesions are mostly uniformly enhanced, while malignant lesions are mostly non-uniformly enhanced. The morphology after ultrasound enhancement can be used to preliminarily determine the benign or malignant tendency of the lesion (31). The lungs have two blood supply systems, from the pulmonary artery and bronchial artery. Studies have shown that diseases such as pneumonia and atelectasis are supplied by pulmonary blood vessels, while malignant tumors are mainly supplied by bronchial arteries. If the enhancement time after injection of contrast agent is less than 6 s, it indicates pulmonary artery supply, and if it is more than 6 s, it indicates bronchial artery supply. The enhancement time of the pulmonary artery supply area is slightly earlier than that of the bronchial artery supply area. After the contrast agent is injected through the elbow vein, it first appears in the atelectasis tissue, pulmonary consolidation tissue and diseased tissue with pulmonary artery supply through the pulmonary artery. Therefore, malignant tumors often show a later enhancement time than benign tumors. Compared with the surrounding early enhanced atelectasis tissue, the area showing delayed enhancement provides diagnostic value (32).

In summary, CEUS can dynamically display the diameter, course and number of blood vessels in the lesion in real time, avoid respiratory interference, guide the selection of the best puncture path, and reduce the occurrence of complications. Therefore, CEUS can serve as a vital supplementary technique when conventional ultrasound has limitations in assessing the internal structure of a lesion. By imaging with a blood-pool contrast agent, CEUS can effectively identify necrotic areas not visible on conventional ultrasound, ensuring that biopsy samples are obtained from viable tumor tissue. This fundamentally enhances the accuracy and reliability of ultrasound-guided biopsies (33).

## 7 Summary

### 7.1 Comparative analysis

As discussed in the preceding sections, each imaging modality for percutaneous lung biopsy possesses distinct advantages and limitations, making them suitable for different clinical scenarios. To provide a clearer overview and facilitate clinical decision-making, the key characteristics of these techniques are summarized in Table 1.

**Table 1:** Comparison of different imaging techniques for percutaneous lung biopsy.

Imaging technique	Diagnostic accuracy	Pneumothorax incidence rate	Cost	Lesion type suitability	Limitations
CT	64.0% ~ 97.0% (8)	2.4% ~ 60.0% (8)	Moderate	Suitable for the vast majority of intrapulmonary lesions, especially deep lesions, small nodules, or those requiring precise anatomical localization	Radiation exposure; not fully real-time; sensitive to respiratory motion; high risk of pneumothorax for deep lesions
PET/CT	95.0% ~ 99.0% (13)	12.5–13.7% (11, 12)	High	Complex lesions, necrotic lesions, metabolically active lesions	Not used for real-time guidance; may miss low-metabolism tumors; inflammation can cause false positives; high cost
MRI	95.8% ~ 97.0% (19, 35)	12.3–17.4% (19)	High	Suitable for mediastinal, hilar, or lesions close to major vessels, as well as for patients sensitive to radiation	Expensive equipment requiring specialized instruments; time-consuming procedure; contraindicated for patients with certain implants; presence of motion artifacts
Ultrasound	81.8% ~ 98.3% (29, 32)	1.7% ~ 12.8% (26)	Low	Suitable for superficial lesions of the chest wall or pleura, especially when radiation-free guidance or real-time monitoring is required	Core limitation: cannot penetrate aerated lung tissue, only suitable for superficial lesions; highly operator-dependent
CEUS	94.0% ~ 98.9% (29, 36)	1.1% ~ 2.4% (25, 29)	Moderate	Serves as a supplement to ultrasound to improve biopsy accuracy in hypervascular or necrotic lesions	requires contrast injection and has contraindications; more complex operation with strong time sensitivity

### 7.2 Future prospects and challenges

CT-guided percutaneous lung puncture biopsy is widely used in clinical practice, but it is radioactive and not suitable for repeated operations. It also has disadvantages such as high requirements for body position and easy complications. Through PET/CT-guided percutaneous lung puncture biopsy, the metabolic characteristics and anatomical characteristics of the lesion site can be combined to help more accurately determine the nature of the lesion, but PET/CT is expensive and has greater radiation exposure problems. As a new technology for percutaneous lung puncture biopsy, MRI has the advantages of high spatial resolution and no ionizing radiation, but it is greatly affected by breathing and the image quality is not high. Ultrasound-guided percutaneous lung puncture biopsy is simple to operate, has little trauma, no radioactive radiation, good safety, and can dynamically observe the lesion in real time, but conventional ultrasound may be affected by air-containing lung tissue and cannot clearly show the internal structure of the lesion, reducing the accuracy of diagnosis. The development of ultrasound contrast imaging makes up for the shortcomings of conventional ultrasound. It can clearly distinguish between necrotic areas and active areas, significantly improve the success rate of puncture, and reduce the occurrence of complications. In recent years, the advantages of contrast-enhanced ultrasound imaging in guiding percutaneous lung biopsy have become increasingly prominent. However, numerous technical and clinical challenges remain, with its primary limitation being the restricted penetration depth, which hinders the visualization and biopsy of deep-seated or centrally located lesions. Future technological advancements should focus on improving the penetration capability of ultrasound signals and developing high-frequency probes with enhanced resolution for deep tissue.

Furthermore, emerging technologies such as artificial intelligence (AI) assistance and multimodal image fusion navigation systems hold promise for reshaping this field. AI-assisted lesion segmentation and feature extraction in CEUS can standardize the selection of biopsy target areas and reduce operator dependence. The multimodal image fusion navigation system enables precise real-time guided sampling or ablation of lesions by integrating metabolically active region information with high-resolution anatomical images and real-time dynamic visualization, while effectively avoiding critical vascular structures (34).

Although various technologies have relatively clear clinical application pathways, there remain significant gaps to address in the future. Currently, data on complication rates across different image-guided techniques are largely based on short-term studies, and there is a lack of large-scale, long-term comparative research, particularly in high-risk populations such as the elderly or those with poor pulmonary function. Additionally, while CEUS demonstrates significant advantages in evaluating peripheral lesions, its diagnostic efficacy and application value for centrally located lung lesions remain unclear. Prospective clinical trials are urgently needed to validate these aspects and expand its applicability. Moreover, emerging technologies such as AI-assisted lesion analysis and the multimodal image fusion navigation system, though promising, require further validation through high-quality studies to confirm their clinical benefits and cost-effectiveness. Therefore, multidisciplinary collaboration and robust clinical research are essential to optimize biopsy strategies and advance the development of percutaneous lung biopsy techniques.
